# Expiratory flow limitation developed in ICU patients: relationship of fluid overload, respiratory mechanics, and outcome

**DOI:** 10.1186/s13054-019-2723-z

**Published:** 2020-01-24

**Authors:** Heyan Wang, Hangyong He

**Affiliations:** 1 Department of Critical Care Medicine, The Sixth Hospital of Guiyang, Guiyang City, Guizhou Province China; 2 Department of Respiratory and Critical Care Medicine, Beijing Institute of Respiratory Medicine, Beijing Chao-Yang Hospital, Capital Medical University, No. 8 Gongren Tiyuchang Nanlu, Chaoyang District, Beijing, 100020 China

Dear editor,

We read with great interest in the report by Volta and colleagues [1] about the presence and determinant of expiratory flow limitation (EFL) developed in patients admitted to an intensive care unit (ICU). They found that the presence of EFL is common among ICU patients requiring mechanical ventilation for acute respiratory failure of different etiologies. And interestingly, the major determinant for developing EFL in patients during the first 3 days of their ICU stay is a positive fluid balance. However, whether there is a relationship among fluid overload, respiratory mechanics, and outcome is controversial, and we would like to add some comments.

First, in Volta et al.’s work [1], 37 (31%) patients exhibited EFL upon admission, of whom 76%, 57%, and 43% had heart diseases, COPD or ARDS, and higher BMI, respectively. It is easily explainable that obese patients and those with COPD, heart disease, or ARDS can exhibit EFL at ICU admission [2]. Therefore, it should be more important to focus on patients who might develop EFL during the ICU stay and its mechanism.

Second, whether fluid overload is the mechanism of developing EFL during the ICU stay was not fully explained by Volta et al.’s data [1]. A decreased respiratory system compliance and an increased airway resistance should be related to fluid overload-induced pulmonary edema, pleural effusion, or small airway swelling and closure [3]. Of note, in Volta’s study, data of respiratory mechanics of patients developing EFL during the ICU stay were not different from those obtained in the absence of EFL. Thus, it is still unclear why patients with fluid overload might develop EFL during the ICU stay.

The third question is whether EFL or fluid overload has a direct effect on the outcome of ICU patients, especially in patients developing EFL after ICU admission, is unclear. In the study by Volta et al., patients who had EFL at admission and developed EFL had higher ICU mortality compared to those without EFL. But patients with EFL had significantly more severe baseline status (SOFA, mMRC, and NYHA scores) and commodities, which may be related to a high mortality rate. On the other hand, ICU mortality had no difference between patients who developed EFL during the first three ICU days and patients never developed EFL. Although the relationship was found between the EFL development and fluid overload, positive fluid balance during the early period of ICU stay may be more directly related to high mortality [4].

Finally, the use of PEEP was suggested by Volta et al. to avoid EFL. However, higher PEEP may lead to venous return reduction, subsequent hemodynamic depression, and possible higher fluid requirement [5], and cause more positive fluid balance and EFL. Thus, whether increasing PEEP is a suitable management for EFL is uncertain.

Therefore, the relationship of fluid overload, respiratory mechanics, and outcome for ICU patients remains unclear, and further studies are needed to determine whether and why preventing EFL is a target for ICU management.

## Authors’ response

C. A. Volta and S. Spadaro

Thank you for giving us the opportunity to reply to the valuable comments raised by Wang et al. regarding our article [1].

As pointed out by Wang et al., patients developing expiratory flow limitation (EFL) during their ICU stay are a subgroup of patients that deserve great attention since the possible role played by a positive fluid balance on the development of EFL. The authors were skeptical of the relationship between a positive fluid balance and EFL, mainly because cumulative fluid balance greater than 10% was not associated with variation of respiratory mechanics. However, EFL is dynamic phenomena pertaining only to expiration, and it is due to a narrowing of the airways when the pressure outside is higher than the pressure inside the airways. Hence, EFL per se should not be responsible for the increased inspiratory resistance. It is true that patients with EFL usually exhibit higher inspiratory resistance, but this should relate to the chronic lung disease associated with EFL [6]. Furthermore, the fact that the respiratory compliance did not vary between patients with and without EFL reflects the limit of this parameter that represents a mean value across different lung regions unlikely influenced by little variation of lung/airways edema responsible for small airways instability during expiration. Of note, in the study on liberal versus conservative fluid management in acute lung injury patients, the values of the plateau pressure were exactly the same in both groups (26.2 ± 0.4 cmH_2_O) [7].

Patients with EFL exhibited a worse outcome as correctly reported by the authors. Indeed, EFL should reflect the severity of respiratory diseases. Of note, the presence of EFL was the strongest predictor of postoperative pulmonary complications [8]. Hence, it is not surprising that patients with EFL exhibited higher SOFA and MRC/NYHA scores. On the other hand, patients developing EFL during ICU stay have equal outcomes compared to those without EFL. This could be due to the limited number of patients and the fact that these patients had a less severe lung disease compared to those presenting EFL at ICU admission.

Regarding the use of PEEP in the presence of EFL, the presence EFL implies opening/closure of the small airways, which should be regarded as a pro-inflammatory mechanism. Furthermore, it has been previously demonstrated that the PEEP able to avoid EFL was associated with both better oxygenation and lung function [9].

## Data Availability

Not applicable.

## Abbreviations

Cst,rs: Static compliance, respiratory system
EFL: Expiratory flow limitation
ICU: Intensive care unit
mMRC: Modified Medical Research Council
NYHA: New York Heart Association Classification
PEEP: Positive end-expiratory pressure
